# Surgical protocol for pulmonary artery banding in mice to generate a model of pressure-overload-induced right ventricular failure

**DOI:** 10.1016/j.xpro.2023.102660

**Published:** 2023-10-25

**Authors:** Argen Mamazhakypov, Christine Veith, Ralph Theo Schermuly, Akylbek Sydykov

**Affiliations:** 1 Department of Internal Medicine, Member of the German Center for Lung Research (DZL), Justus Liebig University of Giessen, Giessen, Germany

**Keywords:** Cell Biology, Health Sciences, Model Organisms

## Abstract

Right ventricular failure (RVF) is the leading cause of death in patients with pulmonary hypertension. Here, we present a protocol for pulmonary artery banding in mice to generate a model of pressure-overload-induced RVF. We describe steps for anesthesia of mice, endotracheal intubation, and pulmonary artery banding surgery. We then detail procedures for phenotyping and analysis. Our approach does not involve complete blockage of the pulmonary flow during clip placement and is, therefore, associated with low intraoperative mortality.

For complete details on the use and execution of this protocol, please refer to Veith et al. (2020).^1^

## Highlights

• Steps for mouse anesthesia and orotracheal intubation for RVF model
• Step-by-step guide for pulmonary artery banding
• Evaluation of right ventricular function and structure

## Abstract (editor highlights)

Publisher’s note: Undertaking any experimental protocol requires adherence to local institutional guidelines for laboratory safety and ethics.

## Graphical abstract

**Figure:**
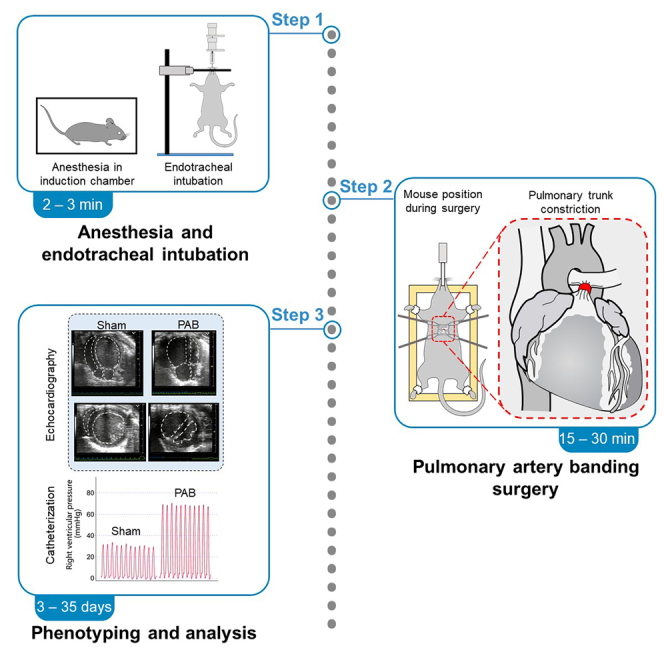

## Before you begin

### Institutional permissions

Male Black Swiss mice (20–30 *g*) were used in these experiments (Charles River Laboratories, Sulzfeld, Germany). Mice were given free access to water and food and were housed under a controlled temperature and standard light-dark cycle (12-h/12-h) throughout the experimental period. All experiments were approved by the governmental ethics committee for animal welfare (Regierungspräsidium Giessen (GI 20/10 Nr. 39-2012), Germany).

### Preparation of home-made customized instruments

**Timing: 1–2 h**

**Figure 1:**
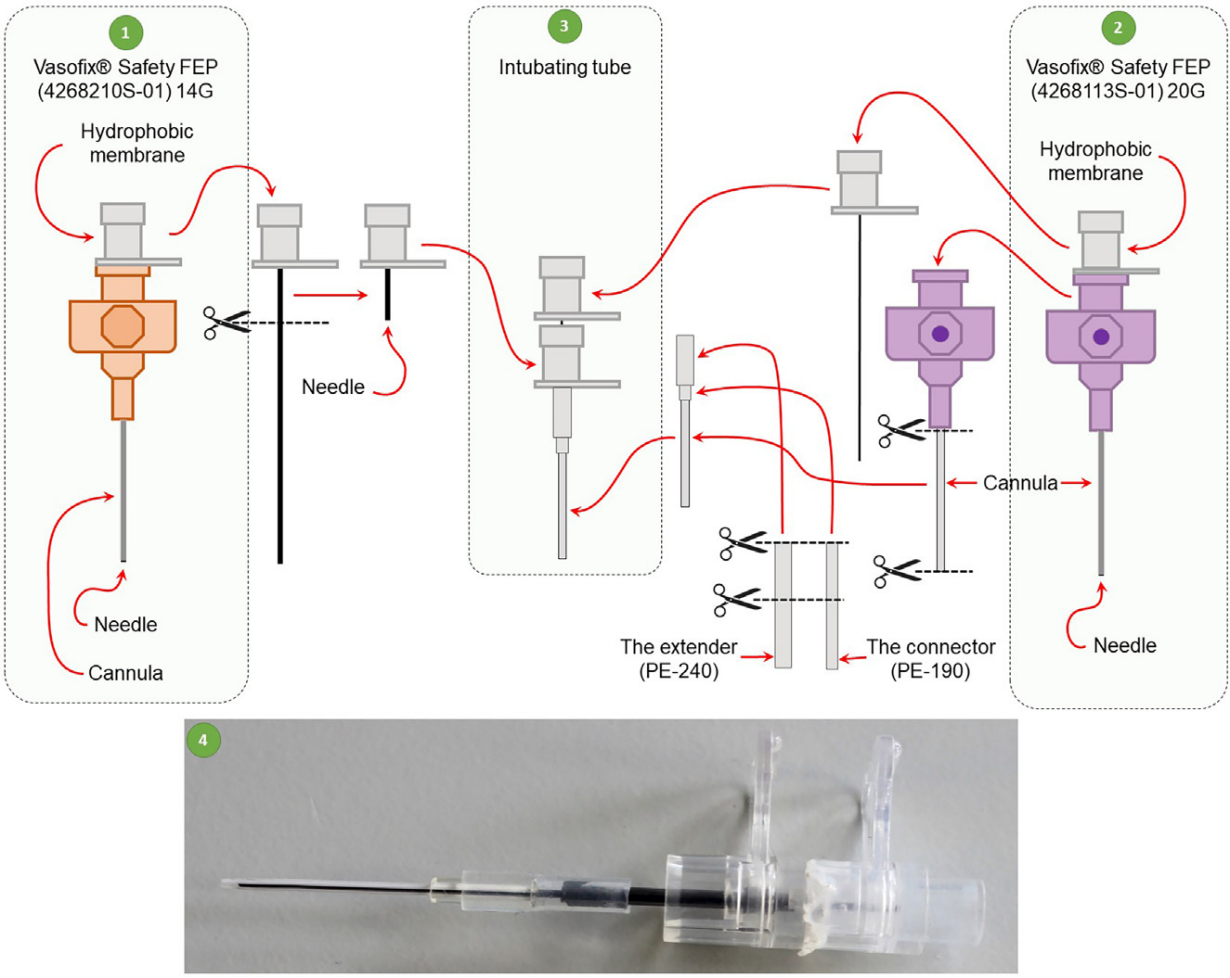
Schematic representation of the preparation of the homemade mouse endotracheal tube 1) 14-gauge intravenous catheter, 2) 20-gauge intravenous catheter, 3) Endotracheal tube made up from the components of 14-gauge and 20-gauge catheters, 4) photograph of the endotracheal tube.

***Note:*** The 7.0-cm-long stylet serves as a stiff guide to facilitate the introduction of the endotracheal tube into the trachea.

***Note:*** The main advantage of this endotracheal tube construction is that the fingers do not block the view during insertion of the tube into the trachea because they are placed not on the tube itself, but on the needle grips. In addition, this construction is much easier in handling than a single thin tube.

1. Preparation of a customized mouse endotracheal tube construction (Figure 1).
  a. Cut off a 2.8 cm long piece of the catheter tube of the intravenous 20-gauge catheter (Cat#10309), which will serve as an endotracheal tube.
  b. Cut off the sharp part of the cannula of the intravenous 14-gauge catheter (Cat#4268210S-01) to create a 3 cm long blunted cannula containing the needle hub.
  c. Cut off the sharp tip of the cannula of the intravenous 20-gauge catheter to create a 7 cm long stylet containing the needle hub.
  d. Attach the endotracheal tube to a 0.8 cm long PE-190 tubing serving as a connector.
  e. Attach the endotracheal tube with the connector to a 1.5 cm long PE-240 tubing serving as an extender that connects the endotracheal tube via a Y-shaped connector to the ventilator.
  f. Attach the endotracheal tube with the connector and extender to the 3 cm long blunted 20-gauge cannula (see step 1b).
  g. Insert a 7.0-cm-long 20-gauge stylet (see step 1c) into the endotracheal tube through the attached blunted 14-gauge cannula.

2. Preparation of a customized mouse vessel probe (Figure 2.1).
  a. Bent the tip of a 26-gauge blunted needle to make an L-shape.
  b. Attach the vessel probe to a 2-mL syringe serving as a holder.

**Figure 2:**
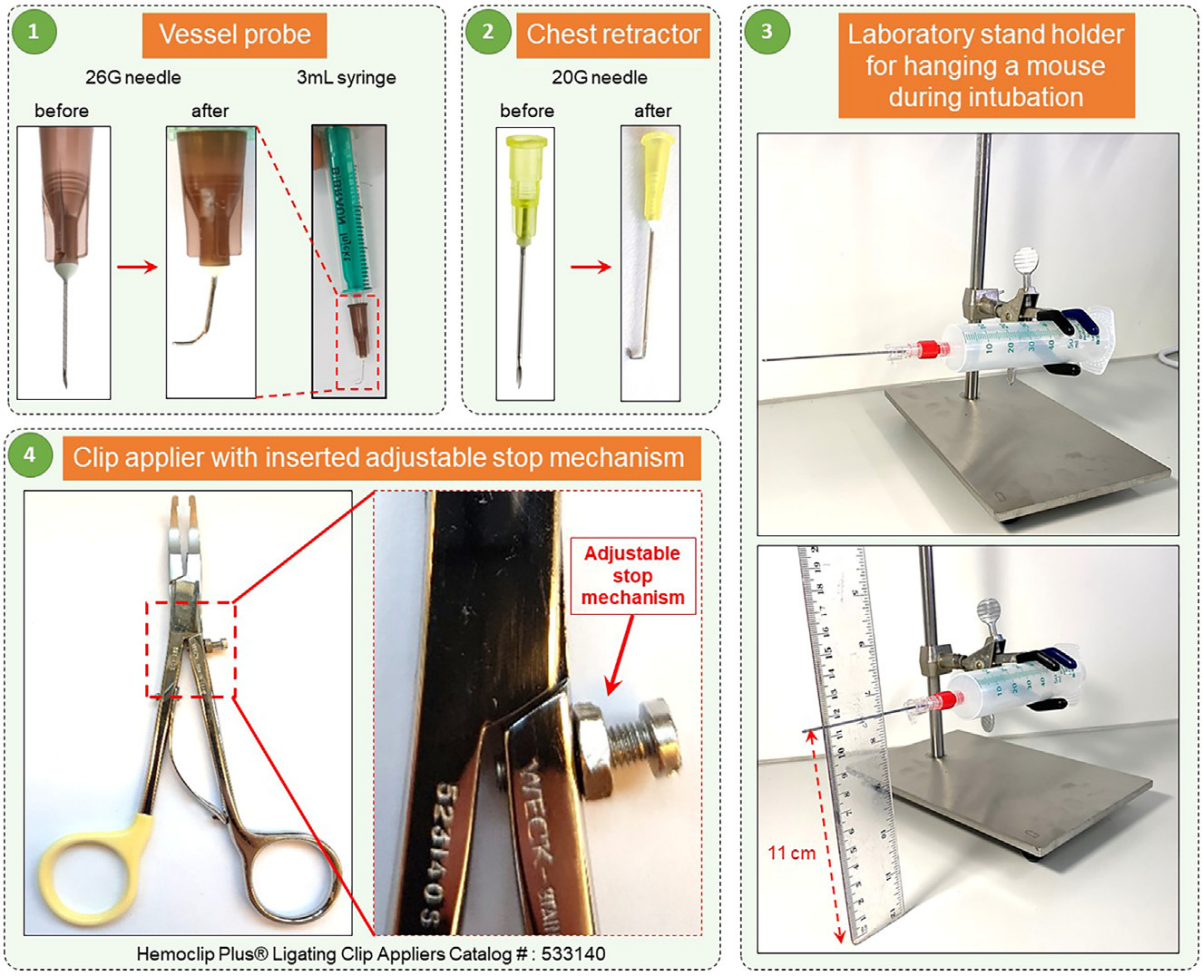
Preparation of the customized instruments 1) Vessel probe made from a 26-gauge needle by blunting and curving its tip, 1) Chest retractor made of 20-gauge needles by blunting, flattening, and curving their tips, 4) Adjustable stop mechanism is fixed to set a required degree of clip constriction, thus yielding reproducible banding severity, 3) Intubation stand holder used to suspend the mouse during intubation.
3. Preparation of customized mouse chest retractors (Figure 2.2).
  a. Cut off the sharp tips of 20-gauge needles.
  b. Flatten the blunted 20-gauge needles.
  c. Bend the flattened needles twice into shapes as shown in Figure 2.2.
4. Customization of the clip applier (Hemoclip, Cat# 523140) by installing an adjustable stop mechanism (Figure 2.4).
  a. Make a hole in one of the handles of the clip applier.
  b. Insert into the hole a small bolt and fix it with a nut of the corresponding size.

***Note:*** Adjustment of the required stenosis degree for the pulmonary artery banding is achieved by changing the level of insertion of the bolt and thus the distance between the handles, so that upon full closure the applier tips are uniformly apart. Needles of various sizes are used as references for the degree of the clip constriction.

5. Preparation of a customized mouse intubation stand (Figure 2.3).
  a. Fix a 50-mL syringe (Cat#8728810F) horizontally to a laboratory stand using a clamp.
  b. Adjust the height of the clamp at around 10–12 cm from the bottom.
  c. Attach blunted 18-gauge needle to the syringe.

**Figure 3:**
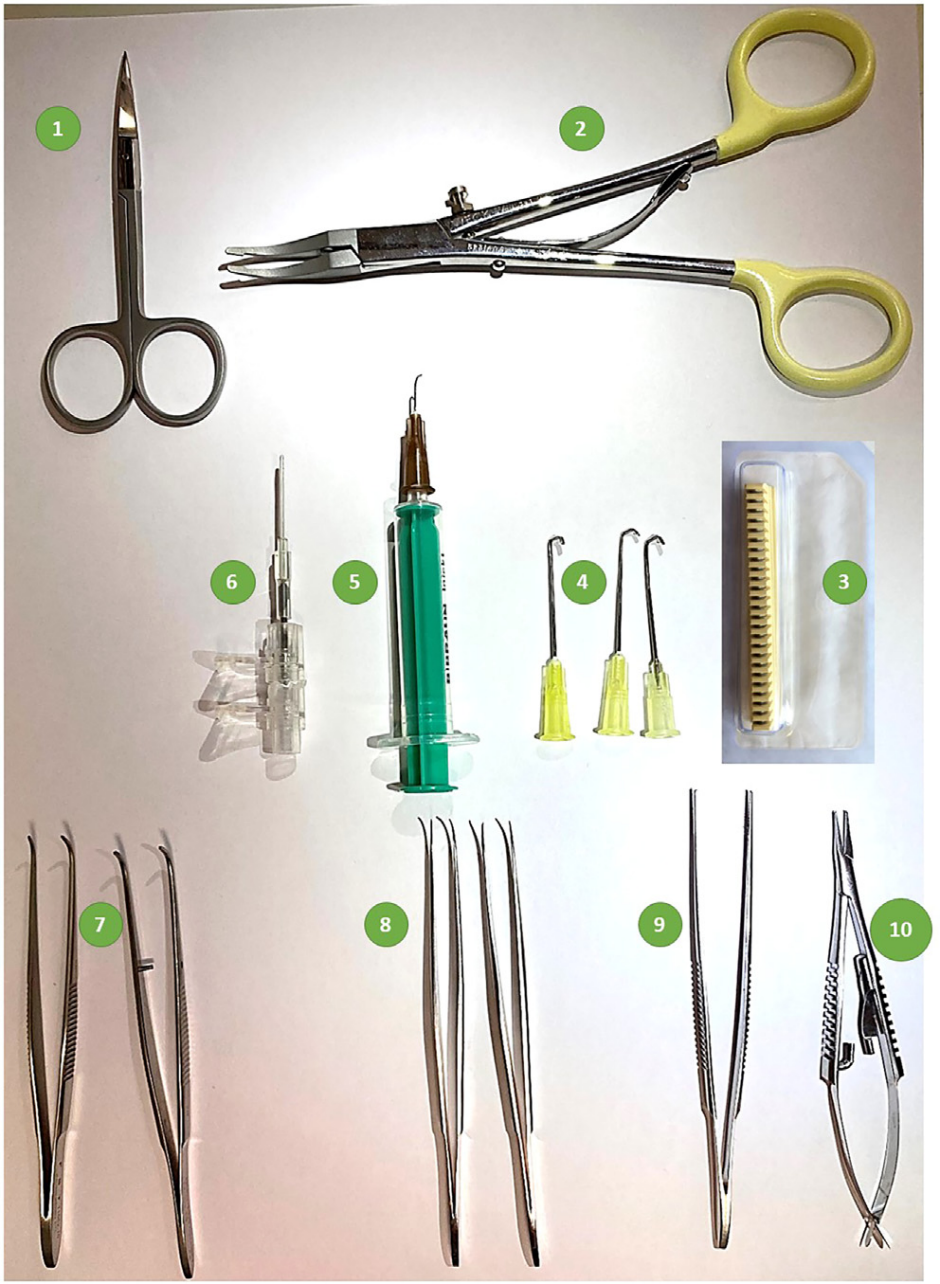
Instruments required for PAB surgery 1) Fine Scissors - CeramaCut, 2) Hemoclip Traditional ligating clip applier, 3) WECK 523735 Hemoclip Traditional small titanium ligating clips, 4) Chest retractors, 5) Vessel probe, 6) Endotracheal tube, 7) Graefe Forceps, curved, 8) Moria Iris Forceps, curved. 9) Graefe Forceps, straight, 10) Castroviejo Micro Needle Holder.

***Note:*** Prepare all these instruments in advance of PAB surgery experiments (at least one day before). Once prepared, these instruments including endotracheal tube, chest retractors, vessel probe, and intubation stand can be cleaned and reused multiple times (Figure 3).

## Key resources table

**Table:** 

REAGENT or RESOURCE	SOURCE	IDENTIFIER
**Chemicals, peptides, and recombinant proteins**		

Isoflurane	Baxter	Cat# HDG9623
Helizyme	B. Braun Melsungen	Cat# 18767
Physiological saline solution	B. Braun Melsungen	Cat# 3570310
Braunoderm	B. Braun Melsungen	Cat# 3881105
Buprenorphine (Temgesic)	Essex Pharma	NA

**Experimental models: Organisms/strains**		

Mouse: wild-type Black Swiss; 8–12 weeks old; male	Charles River Laboratories	Cat# Crl:NIHBL(S)

**Software and algorithms**		

GraphPad Prism 9	GraphPad	https://www.graphpad.com/scientific-software/prism/
LabChart 7	ADInstruments	https://www.adinstruments.com/products/labchart
VevoLab	FUJIFILM VisualSonics	https://www.visualsonics.com/product/software/vevo-lab

**Other**		

Prolene 6-0	Prolene, Ethicon	Cat# EH7228H
Intravenous Vasofix Safety catheter (20Gx1¼”, 1.1 × 33 mm)	B. Braun Melsungen AG	Cat#10309
Intravenous Vasofix Safety catheter (14Gx2″, 2.2 × 50 mm)	B. Braun Melsungen	Cat#4268210S-01
Needle (26Gx1″, 0.45 × 25 mm)	B. Braun Melsungen	Article#4657683
Needle (20Gx1½”, 0.90 × 40 mm)	B. Braun Melsungen	Article#4657519
Perfusor Syringe, 50 mL	B. Braun Melsungen	Cat#8728810F
Injekt Luer Solo Syringe, 2 mL	B. Braun Melsungen	Cat#4606027V
PE-190 tubing		NA
PE-240 tubing		NA
Castroviejo Micro Needle Holder –Straight w/Lock 9cm	Fine Science Tools	Cat# 12060-01
Hardened Fine Scissors, straight	Fine Science Tools	Cat# 14090-11
Moria Iris Forceps, curved	Fine Science Tools	Cat# 11370-31
Graefe Forceps, straight	Fine Science Tools	Cat# 11050-10
Graefe Forceps, curved	Fine Science Tools	Cat# 11052-10
Clip applier	Hemoclip, Edward Weck, Research Triangle Park	Cat# 523140
Small titanium ligating clip	Hemoclip, Edward Weck, Research Triangle Park	Cat# 523735
Dexpanthenol eye ointment Bepanthen	Bayer Vital GmbH	NA
Adhesive tape		NA
Homeothermic Controller and Plate (for mice)	AD Instruments	Cat# ML295/m
Laboratory Stand with a clamp	Carl Roth	NA
Germinator 500 Glass Bead Sterilizer	Cell Point Scientific	Cat# 720127
Surgical stereomicroscope M50with small boom stand	Leica Microsystems	https://www.leica-microsystems.com/products/light-microscopes/stereo-microscopes/p/leica-m80/
Small-animal ventilator	MiniVent type 845, Hugo Sachs Elektronik	Cat# 73-0044
Anesthesia system	Tabletop Laboratory Animal Anaesthesia System, VetEquip Inc	Cat# 901806
A power light with flexible horns KL-200	Fiber Optics Schott	https://www.schott.com/de-de/products/kl-fiber-optic-light-sources-p1000348
Vevo 770	FUJIFILM VisualSonics	https://www.visualsonics.com/product/imaging-systems/vevo-770

***Alternatives:*** References for basic laboratory materials, equipment, and surgical tools, in the key resources table, can be replaced by similar products from other providers.

## Step-by-step method details

### Preparation of instruments and animals

**Timing: 5 min**

This section details the necessary pre-procedural analgesia and sterilization of the surgical instruments.

1. Sterilize the surgical tools in Germinator 500 Glass Bead Sterilizer (Cat# 720127) before surgery.

***Note:*** Clean all instruments thoroughly with an enzymatic detergent (Helizyme; Cat# 18767) after each procedure and between surgeries on different animals. Remove tissue remnants left on the surgical instruments using a soft brush.

2. Inject subcutaneously an analgesic buprenorphine hydrochloride (Temgesic, 0.1 mg/kg) 30 min prior to surgery.

**Pause point:** 30 min.

### Mouse anesthesia and orotracheal intubation

**Timing: 2–3 min**

This section encompasses anesthesia induction and orotracheal intubation in mice. After induction of anesthesia, a stable level of anesthesia is maintained for all subsequent steps of the surgical procedure.

3. Place the mouse into a transparent anesthesia induction chamber (Tabletop Laboratory Animal Anesthesia System, VetEquip, Cat# 901806) supplied with a continuous flow of isoflurane (3%–3.5% in 100% oxygen at a flow rate of 1 L/min, Cat# HDG9623).
4. Ensure proper depth of anesthesia by performing a toe pinch with straight forceps (Graefe Forceps, straight, Cat# 11050-10) after the animal has lost its righting reflex.
5. Upon achievement of a required depth of anesthesia, take the mouse out of the box and suspend it by hooking its superior incisors over a blunt needle on the intubation stand (Figures 4.2–4.4).

**Figure 4:**
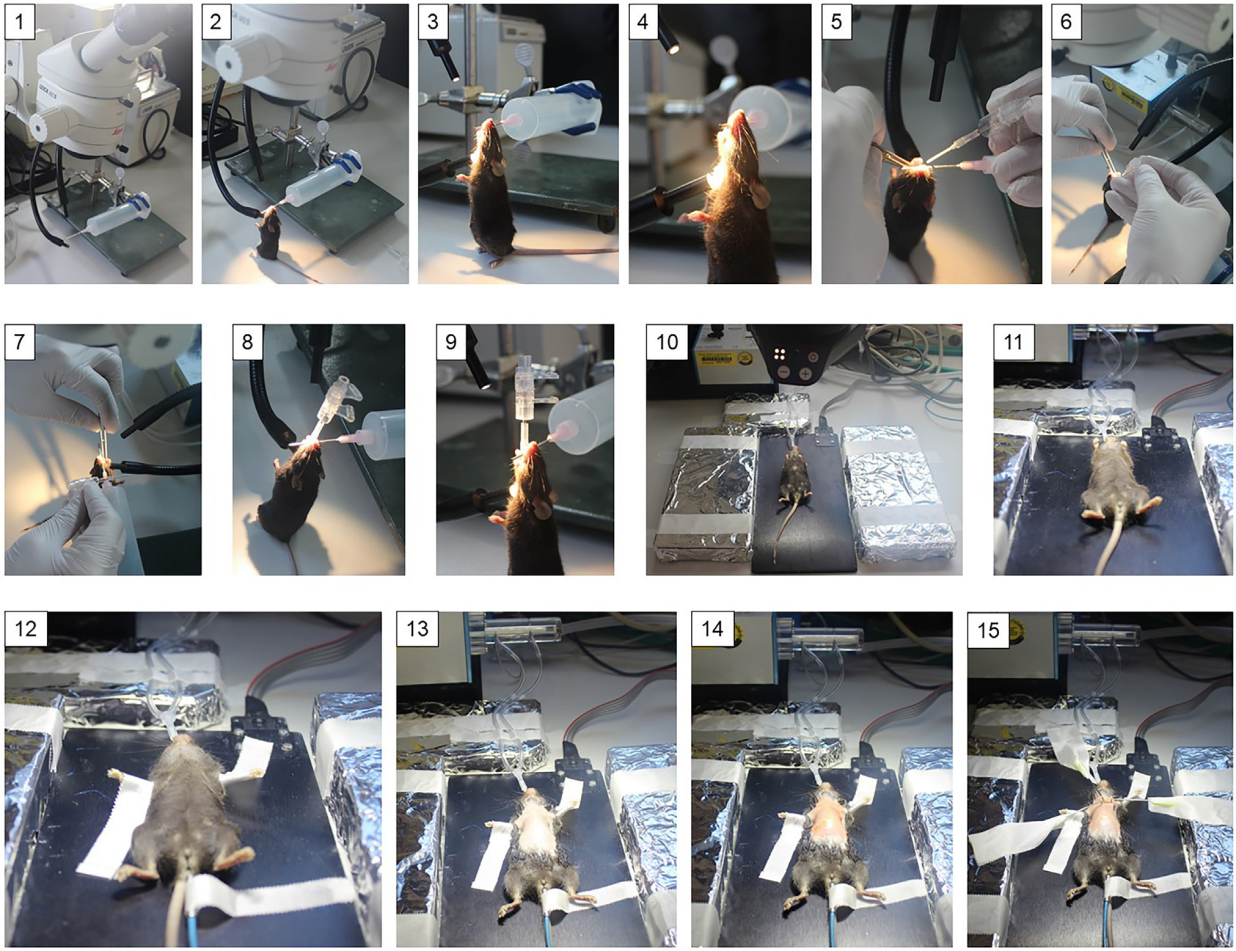
Sequence of steps from mouse intubation to fixation on the surgery plate (1) Surgical stereomicroscope, customized intubation stand, and power light with flexible horns. (2, 3, 4) Suspension of the mouse over the needle of the intubation stand. (5, 6, 7) Insertion of the endotracheal tube into the mouse trachea. (8, 9) Successful insertion of the intubation tube into the mouse trachea. (10, 11) Placement of the mouse on the homeothermic plate following intubation and connection to the ventilator. (12) Fixation of the limbs to the homeothermic plate and insertion of a rectal thermal probe. (13) Shaving of the chest region. (14) Braunoderm application over the chest skin. (15) Insertion of chest retractors.
6. Use a power light with flexible horns KL-200 for the insertion of the endotracheal tube.

***Note:*** To better visualize the vocal cords and the epiglottis of the mouse, illuminate the ventral neck of the mouse from the outside with one horn and the throat from above using another horn.

7. Under the surgical stereomicroscope at 8x magnification (Leica, M50), retract gently the tongue of the mouse to one side with a curved tweezer (Graefe Forceps, curved, Cat# 11052-10) held in the right hand, while with the left hand widen the oral cavity using a second curved tweezer (Graefe Forceps, curved, Cat# 11052-10) to visualize vocal cords and the epiglottis.
8. Once the vocal cords and the epiglottis are clearly seen, insert the endotracheal tube gently into the trachea with the right hand until the tip of the tube passes between the vocal cords and enters the trachea (Figures 4.5–4.7).
9. Place the mouse in the supine position on a homeothermic plate (AD Instruments, Cat# ML295/m). Remove the extender and connect the endotracheal tube via the Y-shaped connector to the small-animal ventilator (MiniVent type 845, Cat# 73-0044) (Figures 4.10 and 4.11).
10. Evaluate the success of the intubation by visual observation of the chest excursion synchronous with the ventilator.

***Note:*** Calculate the ventilation settings for a mouse using the formulas provided by the Harvard Apparatus:

Tidal volume (mL) = 0.0062 × M^1.01^ and ventilation rate (breaths/minutes) = 53.5 × M^-0.26^, where M is animal body weight in kg.

**CRITICAL:** If the abdomen becomes enlarged with each inspiration stroke, suspect an esophageal intubation. In this case, remove immediately the endotracheal tube, and repeat the intubation procedure according to steps 1‒6.

11. Tape the paws of the mouse down to the homeothermic plate using adhesive tapes.
12. Protect the eyes of the mouse from drying by applying dexpanthenol eye ointment.

***Note:*** The anesthesia is maintained with isoflurane (1.5%–2% in 100% oxygen).

### The surgical procedure of pulmonary artery banding

**Timing: 15–30 min**

The following steps describe the technique of pulmonary artery banding in mice. Pulmonary artery banding is a surgical procedure, in which a titanium clip is placed around the pulmonary trunk to induce constriction of the pulmonary artery.

13. Shave the mouse chest wall and then apply Braunoderm (Cat# 3881105) (Figures 4.14, 5.2a, and 5.2b).

**Figure 5:**
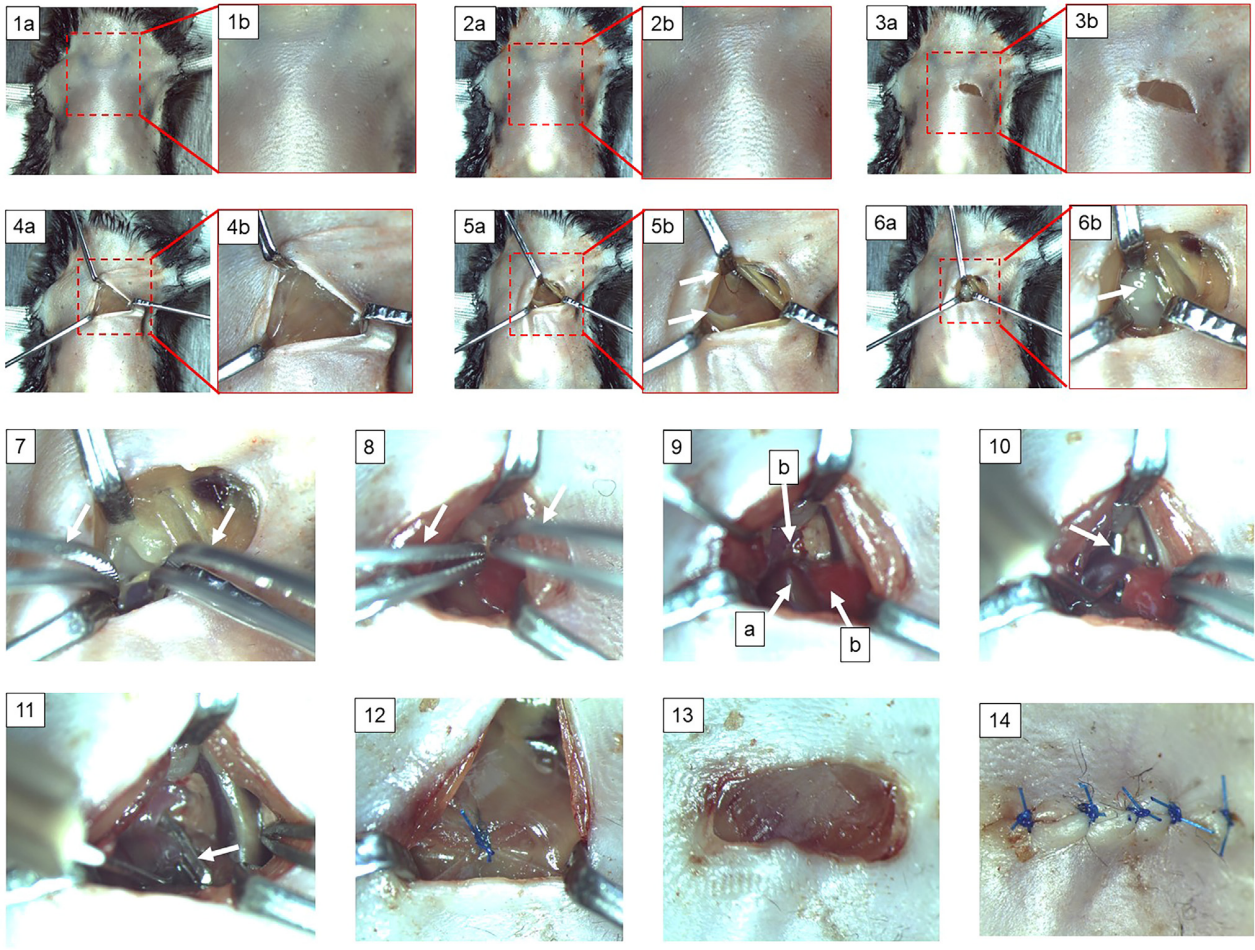
Roadmap of key steps in the protocol for mouse pulmonary artery banding surgery (1a, 1b) Removal of chest hair. (2a, 2b) Braunoderm application over the chest skin. (3a, 3b) Skin incision on the chest. (4a, 4b) Insertion of chest retractors. (5a, 5b) Pulling of chest muscles with chest retractors. A rib (white arrow) and intercostal muscles are visible. (6a, 6b) Pulling apart the ribs with chest retractors. Adipose tissue and thymus (depicted by white arrow) are exposed. (7) Dissection of pericardial fat and connective tissues with curved Moria forceps (white arrows). (8) Isolation of the pulmonary artery using curved Moria forceps (white arrows). (9) Important intrathoracic structures: pulmonary trunk (arrow a), left atrium (arrow b), and aortic arch (arrow c). (10) Creation of a tunnel underneath the pulmonary trunk using the vessel probe (white arrow). (11) Placement of the clip around the pulmonary trunk (white arrow). (12) Closure of the chest cavity by bringing together the adjacent ribs with a suture. (13) Repositioning of the chest muscles to their physiological positions and removal of chest retractors. (14) Skin suturing.

***Note:*** Illuminate the surgical field with the power light with flexible horns throughout the procedure, (Figure 4.12).

14. Make a transverse skin incision (≈ 5 mm) with a scalpel, 2 mm away from the left sternal border and 2 mm lower than the level of the armpit (Figures 5.3a and 5.3b).
15. Gently pull apart the thoracic muscles with retractors to expose the ribs (Figures 5.5a and 5.5b).
16. Make a small incision with scissors (Cat# 14090-11) at the level of the second intercostal space 2–3 mm from the left sternal border to open the thoracic cavity (Figures 5.6a and 5.6b).
17. Insert 3-4 retractors gently into the incision one by one subsequently pulling away to spread carefully the wound 4–5 mm in width (Figure 5.7), then fix to the plate with surgical tapes.
18. Retract the thymus and fat out of the field of dissection.
19. Pull gently the pericardial sac apart using two forceps and attach to the teeth’s of the retractors. After mobilization of the pericardium, the pulmonary trunk can be visualized.
20. Bluntly dissect the pulmonary trunk from the aorta and left atrium using curved Moria forceps (Cat# 11370-31) by carefully disrupting connective and fatty tissues (Figures 5.8 and 5.9).

***Note:*** Make sure not to damage the great vessels (aorta and pulmonary trunk), the left superior vena cava, the left atrium, or the apex of the left lung when dissecting the tissues around the pulmonary trunk.

**Figure 6:**
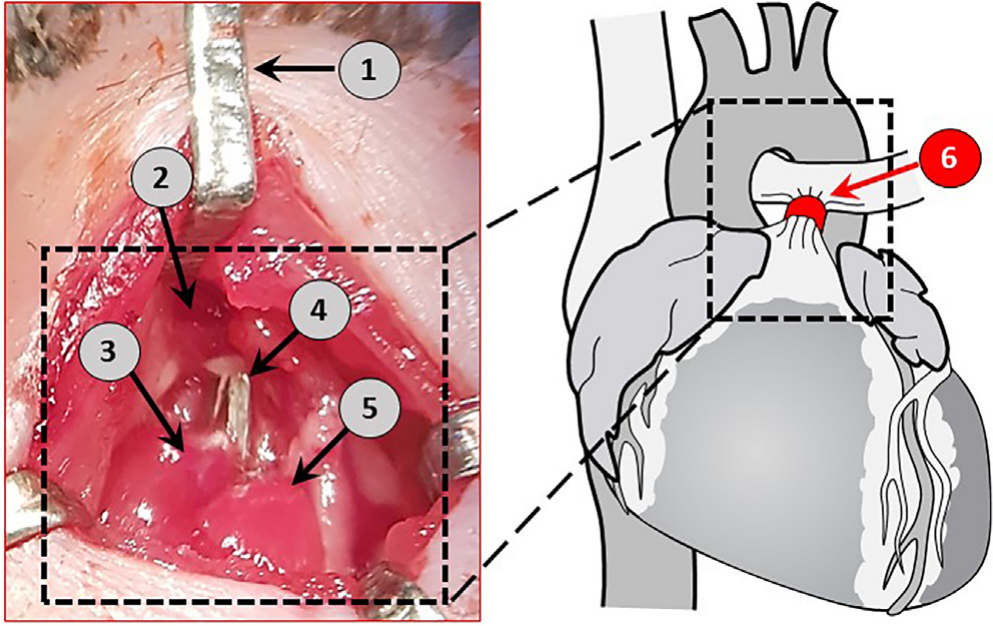
Intraoperative illustration of the pulmonary artery banding 1) chest retractors, 2) aortic arch, 3) pulmonary trunk, 4) ligating clip placed around the pulmonary trunk, 5) left atrium, 6) a schematic representation of the pulmonary trunk constriction.

21. Create a tunnel underneath the pulmonary trunk (Figure 5.10) with an L-shaped vessel probe (Figure 2.1).
22. Place the tip of the vessel probe from the side of the pulmonary trunk closest to the infundibulum and push gently underneath the pulmonary trunk until its end appears between the pulmonary artery and aorta.
23. Place a small Hemoclip titanium ligating clip (Cat# 523735) around the pulmonary trunk with a specially adapted applier set to a width of 0.3 mm in diameter, which corresponds to approximately 65%–70% occlusion of the luminal diameter (Figures 5.11 and 6).

**CRITICAL:** Once the clip is placed around the pulmonary trunk, inspect it to ensure that the ligating clip is constricted and placed properly around the pulmonary trunk.

***Note:*** For the sham operation, dissection of the pulmonary trunk is performed using the vessel probe, but a ligating clip is not placed around the pulmonary trunk.

24. Remove the chest retractors and place the thymus back to its physiological position.
25. Re-inflate the lungs by shutting off the outflow tube on the ventilator for 1–2 s using fingers.
26. Close the chest cavity by bringing together the second and third ribs with a 6-0 polypropylene suture (Cat# EH7228H).

***Note:*** While making a knot, a slight pressure is applied on the chest with the needle holder (Cat# 12060-01) to reduce the volume of free air in the chest cavity (Figure 5.12).

27. Move all muscles back to their physiological positions.
28. Close the skin with 6-0 polypropylene sutures (Figure 5.13).
29. Treat the surgical wound with Braunoderm (Figure 5.14).
30. Turn off the isoflurane.

### Post-operative procedures and follow-up

**Timing: 3–35 days**

This section describes procedures on postoperative care including pain control, and follow-up of mice. A proper care and monitoring of the animals after the surgery are essential for their postoperative recovery.

31. Remove adhesive tapes from the paws.
32. Stop the ventilator temporarily to confirm whether the spontaneous breathing is resumed by checking the regular movements of the chest.
33. Switch off the ventilator once spontaneous breathing is restored and extubate the mouse.
34. Place the mouse under a heating lamp and closely monitor it for the next 4–6 h to ensure a complete recovery from the anesthesia.
35. Keep the mouse in a standard rodent cage with free access to food and water.

**CRITICAL:** Provide postoperative analgesia by administration of buprenorphine hydrochloride (Temgesic, 0.1 mg/kg body weight subcutaneously) two times a day for 3–5 days.

## Expected outcomes

**Figure 7:**
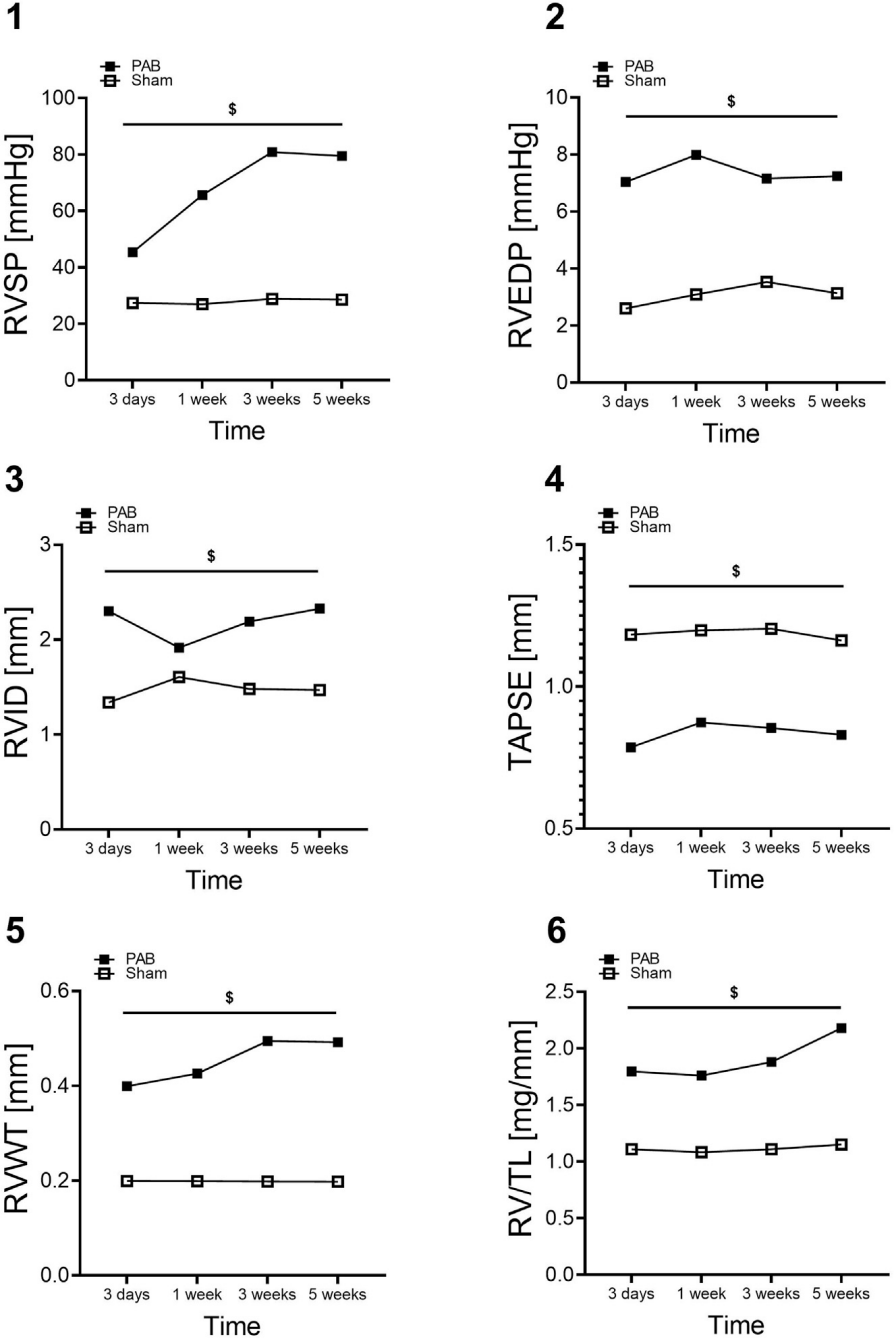
Evaluation of right ventricular geometry and function by echocardiography and right ventricular catheterization (1) Right ventricular systolic pressure (RVSP) measured by right ventricular catheterization. (2) Right ventricular end-diastolic pressure (RVEDP) measured by right ventricular catheterization. (3) Right ventricular internal diameter measured by echocardiography. (4) Tricuspid annulus systolic excursion (TAPSE) measured by echocardiography. (5) Right ventricular wall thickness (RVWT) measured by echocardiography. (6) The ratio of right ventricular mass to tibia length (RV/TL). Data are presented as mean ± standard error of mean (n = 8–15). A two-tailed unpaired Student’s t test was used to compare the differences between the groups and a p-value of <0.05 was considered statistically significant. $Significant differences between sham and PAB. Reprinted and modified from Veith et al.^1^

We evaluated RV function, geometry, and hemodynamics by echocardiography (Vevo 770) and right ventricular catheterization in sham and PAB mice 3 days, 1, 3 and 5 weeks post-surgery (Figure 7). RV hemodynamics were assessed by measurement of right ventricular systolic and end-diastolic pressures (RVSP, RVEDP) during RV catheterization (Figures 7.1 and 7.2). RV remodeling was assessed by measurement of the right ventricular internal dimension (RVID) and right ventricular wall thickness (RVWT) by means of echocardiography (Figures 7.3 and 7.5). The RVID was measured as the distance from the RV free wall to the septum using the apical four-chamber view. The RV free wall thickness (RVWT) was measured in the modified parasternal long-axis view. To assess RV systolic function, tricuspid annulus plane systolic excursion (TAPSE) was measured as the amplitude of the tricuspid valve plane excursions using the apical four chamber view (Figure 7.4). We determined the ratio of RV mass to tibia length for quantitative estimation of RV hypertrophy (Figure 7.6). These data indicate that PAB induces significant RV remodeling and dysfunction, which develops as early as 3 days post-surgery and sustains for the next 5 weeks.

## Limitations

Due to the variations in the pulmonary artery diameter among different mice, it might not always be possible to achieve the same degrees of constriction in all mice. This might lead to a certain degree of variability of the degree of pulmonary artery constriction across mice and can influence the reproducibility of the results. In order to minimize the variability in the stenosis degree, we recommend the following: 1) gaining experience in using the clip applier properly before starting animal experiments to ensure reproducible and comparable degree of clip constriction; 2) selection of mice of comparable age and body weight for experiments to ensure the comparable diameter of the pulmonary trunk in mice; 3). collection of clips after organ harvesting in the end of the experiment to control the appropriate degree of clip constriction in individual animals.

## Troubleshooting

### Problem 1

Esophageal intubation as a potential cause of death during surgery (mouse anesthesia and orotracheal intubation, step 8).

### Potential solution

To avoid esophageal intubation, you should confirm the epiglottis as a landmark of orotracheal intubation. Ensure sufficient illumination of the neck area to clearly visualize the landmarks. Always control the success of intubation by observing the thorax movements following the connection of the intubation tube to the ventilator. When the chest movements synchronous with the ventilator are absent and the abdomen is getting larger with each inspiration stroke, esophageal intubation is highly suspected, and you should immediately extubate the mouse.

### Problem 2

Bleeding associated intra and perioperative mortality (the surgical procedure of pulmonary artery banding, steps 14, 16, 20‒23 and 26). Bleeding can be caused by damage of the subcutaneous veins, left vena cava superior, left atrium, or pulmonary artery.

### Potential solution

Use blunt-ended instruments within the operation field.

### Problem 3

Pneumothorax associated perioperative mortality (the surgical procedure of pulmonary artery banding, steps 16, 20 and 26). Pneumothorax can be caused by damaging the trachea or left lung during the surgery. The trachea can be damaged with sharp instruments during dissection of the pulmonary artery. The left lungs can be damaged with sharp instruments during the thorax opening or with a suturing needle during the chest closure.

### Potential solution

Use blunt-ended instruments as much as possible and avoid damaging the lungs during suturing the ribs by carefully inspecting the area underneath the ribs.

### Problem 4

Inadequate anesthesia (mouse anesthesia and orotracheal intubation, at all steps; the surgical procedure of pulmonary artery banding, at all steps).

### Potential solution

Ensure to prepare the correct dosage of buprenorphine hydrochloride working solution from a stock solution. In addition, monitor the depth of anesthesia and adjust isoflurane concentration according to the mouse response (heart rate, pinch toe reflex).

### Problem 5

Bradycardia (mouse anesthesia and orotracheal intubation, at all steps; the surgical procedure of pulmonary artery banding, at all steps). Anesthesia can cause severe bradycardia, which can compromise the blood supply to the brain. In addition, significant manipulation in the area of great vessels can induce or aggravate bradycardia.

### Potential solution

Carefully monitor heart rate in mice during the surgery. Avoid significant manipulation of the vessels. We recommend ventilation of mice with 100% oxygen during the surgery.

### Problem 6

Acute right heart failure (the surgical procedure of pulmonary artery banding, steps 22 and 23). Lifting the pulmonary artery too much and for a longer time during dissection of the surrounding tissues and during constriction of the clip can cause a temporary interruption of blood flow through the pulmonary artery, increase the afterload on the right ventricle, and cause acute right heart failure and death. Another cause of acute perioperative RV failure is excessive constriction of the pulmonary trunk.

### Potential solution

Lift the pulmonary artery gently and for a very short time without a complete obstruction of the blood flow. To prevent excessive constriction of the pulmonary trunk, it is important to fix the stop mechanism of the clip applier firmly.

### Problem 7

Hypovolemia (the surgical procedure of pulmonary artery banding, at all steps). Due to their small body size, rodents are prone to significant fluid loss especially through the surgical area. Hypovolemia can cause hemodynamic instability and death of the animal.

### Potential solution

We recommend starting fluid support in the beginning and not as a reconstitution in the end of the surgery. Fluids (0.9% NaCl, glucose) can be given subcutaneously divided into small doses or by continuous infusion to prevent acute hypervolemia.

### Problem 8

Hypothermia or hyperthermia (the surgical procedure of pulmonary artery banding, at all steps). Rodents are especially prone to hypothermia during anesthesia because of their high metabolic rate and large surface-to-body-weight ratio. Hyperthermia is also a risk if an active heating device is used. Hypothermia and hyperthermia during the surgery can increase the side effects in mice. It is very important that the target body temperature during anesthesia is maintained in order to avoid adverse physiological consequences of hypo- and hyperthermia. Hypothermia can prolong recovery from anesthesia and can cause myocardial infarctions and coagulopathies, whereas hyperthermia can cause tachycardia, hyperventilation, and cardiac arrhythmias.

### Potential solution

We recommend paying particular attention to monitoring and maintaining body temperature during the surgery.

## Resource availability

### Lead contact

Further information and requests for resources and reagents should be directed to and will be fulfilled by the lead contact, Akylbek Sydykov (akylbek.sydykov@innere.med.uni-giessen.de).

### Materials availability

This study did not generate new unique reagents.

### Data and code availability

This study did not include new datasets.
